# Effect of *Flabellaria paniculata* Cav. extracts on gastric ulcer in rats

**DOI:** 10.1186/1472-6882-12-168

**Published:** 2012-10-02

**Authors:** Margaret O Sofidiya, Lilian Agufobi, Abidemi J Akindele, Johnson A Olowe, Oluwole B Familoni

**Affiliations:** 1 Department of Pharmacognosy, Faculty of Pharmacy, University of Lagos, Lagos, Nigeria; 2 Department of Pharmacology, Faculty of Basic Medical Sciences, College of Medicine, University of Lagos, Lagos, Nigeria; 3 Department of Physiology, Faculty of Basic Medical Sciences, College of Medicine, University of Lagos, Lagos, Nigeria; 4 Department of Chemistry, Faculty of Science, University of Lagos, Lagos, Nigeria

**Keywords:** *Flabellaria paniculata* Cav, Malphighiaceae, Antiulcer, Ethanol-induced ulcer, Indomethacin-induced ulcer, Pylorus ligation-induced ulcer, Acute toxicity

## Abstract

**Background:**

The leaves and root of *Flabellaria paniculata* (Malpighiaceae) are frequently used in the treatment of wounds and ulcers in Nigerian folk medicine. The purpose of this study was to compare the effect of ethanolic extracts from the leaves (FPL) and root (FPR) of *F. paniculata* on gastric ulcers in rats.

**Methods:**

The effect of FPL and FPR (100, 200 and 400 mg/kg) was evaluated in ethanol and indomethacin gastric ulcer models. Control groups for FPL and FPR were orally treated with 3% Tween 20 and distilled water respectively. FPL was further investigated in pylorus ligation model. Misoprostol and cimetidine were used as reference.

**Results:**

FPL significantly (P < 0.05) reduced gastric lesions by 82.22% and 67.32% in ethanol and indomethacin induced ulcer models at 100 mg/kg respectively while FPR (100, 200 and 400 mg/kg) did not exert significant effect in the two models. In pylorus ligation model, FPL exerted a significant preventive antiulcer effect as indicated by reduction in gastric volume at 200 and 400 mg/kg doses. Only 400 mg/kg of the extract exerted a significant reduction in ulcer index when compared with the control group. The oral route LD_50_ of FPL was estimated to be 4570 mg/kg while that of FPR was 2754 mg/kg. The LD_50_ in intraperitoneal injection was estimated to be 1202.26 and 1380.38 mg/kg for FPL and FPR respectively. The phytochemical investigation showed that both extracts possess triterpenoids and saponin, while the presence of flavonoid was detected only in FPL.

**Conclusions:**

The results of this study indicated that FPL and not FPR is effective against experimentally induced gastric ulcers. The presence of varied phytochemical constituents probably influenced the pharmacological differences between the two extracts.

## Background

Increase consumption of alcohol and non-steroidal anti-inflammatory drugs (NSAID) and inappropriate diets have contributed to the growing ulcer etiopathology around the globe. Thus peptic ulcer is considered a disease of modern times related to increasingly frequent addictions and stressful lifestyle [1]. Besides treatment with synthetic drugs or changes in daily routine, such as nutrition and exercise, other interesting option especially in developing countries, is the use of herbal remedies and preparations.

The genus *Flabellaria* includes one variable species and is widely distributed in tropical Africa, in the forest, especially along rivers. *Flabellaria paniculata* Cav. (Malpighiaceae) is a climbing shrub, about 3–15 m high [2]. The lamina of the leaves are broadly elliptic, ovate or rarely lanceolate and the petioles are between 1–2.5 cm long.

The leaves and root of this plant are sold and prescribed as ingredients of herbal preparations in the treatment of wound and ulcers on herbal stalls in Lagos metropolis, Nigeria (informal survey). The leaves are also used in Ghana on wounds and ulcers and as an ecbolic in Côte d'Ivoire [2]. The wound healing and antibacterial property of the leaf has been reported [3,4]. This study was designed to make a comparative study of the antiulcer activity of leaf (FPL) and root (FPR) extracts of *F. paniculata* with the aim of establishing which of these extracts is more effective as an antiulcer agent.

## Methods

### Chemicals and drugs

The following drugs and chemicals were used: Absolute ethanol, chloroform, indomethacin (Sigma Chemical Company, St. Louis, MO, USA); Misoprostol (Cytotec® tablet, Pharmaceutical Laboratories, Germany), Cimetidine (Cetilab-400®, Laborate Pharmaceutical, India), Tween 20, Urethane (Aldrich Chemical Co. Ltd, Gillingham Dorset, England).

### Experimental animals

Adult albino rats and mice of both sexes (100–150 g and 16–30 g respectively) were purchased from the Laboratory Animal Centre, Redeemer University, Ogun State and maintained in the Animal House of College of Medicine, University of Lagos, Nigeria. They were housed in cages and placed on standard pellet feed (Livestock Feed PLC, Ikeja, Lagos, Nigeria) and were given free access to clean water. Each animal was identified by body marks using 1% picric acid solution. They were kept in well ventilated rooms with 12/12 h light/dark conditions and ambient room temperature. Animals were procured two weeks before the experiments to acclimatize with the laboratory environment. The experimental procedures used in this study conform to the United States National Institutes of Health Guidelines for Care and Use of Laboratory Animals in Biomedical Research [5] and approved by the Committee on Ethics in Animal Experimentation of College of Medicine, University of Lagos, Lagos, Nigeria.

### Plant material

The plant materials were collected from Ilaro, Egbado in Ogun State, Nigeria, in July 2010. The plant samples were authenticated by Mr T. K. Odewo of the herbarium unit of Botany Department, University of Lagos. Voucher specimen (LUH 2778) was deposited in the herbaria of the Department of Botany and Department of Pharmacognosy, University of Lagos.

### Preparation of extracts

The leaves were air dried while the root was cut into bits and dried in the oven at 45°C. The dried samples were ground into uniform powder using Christy and Norris 8’ Lab Milling Machine (serial No. 50158). The powdered samples (500 g) were macerated with absolute ethanol (1.5 L) for 48 hours at room temperature with constant shaking. Then, the resulting extracts were filtered over Whatman No. 1 paper and the filtrate evaporated to dryness under vacuum on a rotary evaporator (Heidolph-Rotacool, Germany) at 38°C.

### Phytochemical analysis

Preliminary phytochemical screening and thin layer chromatography (TLC) were used to evaluate the chemical composition of FPL and FPR crude extracts [6]. FPL was eluted with hexane: ethyl acetate (17:3) while FPR was eluted with butanol: acetic acid: water (4:1:5) and revealed with reagents specific chromophores, dragendorff for alkaloid; anisaldehyde for terpenoids and ferric chloride for phenolic compounds.

### Acute toxicity tests

For the assessment of acute toxicity, male and female mice were divided into groups of 5 animals each. FPL and FPR extracts were given p.o. at doses of 50 to 5,000 mg/kg and i.p at doses of 50 to 2000 mg/kg. The control groups received 3% Tween 20 and distilled water (5 ml/kg, p.o.) respectively. The mortality within 24 h period was determined and the LD_50_ estimated by the log dose–probit analysis method [7] using GraphPad Prism software.

### Antiulcer assay

Ethanol, indomethacin and pylorus ligation were used as ulcerogenic agents in this study. The antiulcer potential of the extracts were tested at three doses (100, 200 and 400 mg/kg). Dissolution of the dried extracts in a common vehicle was a challenge; therefore, two negative controls were used; distilled water (5 ml/kg) for FPR extract treated group and 3% Tween 20 for FPL extract treated group. Misoprostol and cimetidine (100 mg/kg) were used as reference drugs. All drugs and extracts were administered orally.

### Ethanol induced gastric ulcer

Anti-ulcerogenic effect of FPL and FPR extracts was investigated first in ethanol-induced ulcer model [8]. Rats were fasted for 24 h prior to the administration of the drugs and extracts. On the day of the experiment, groups 1–6 (FPL and FPR treated group) received 100, 200 and 400 mg/kg of the extracts (p.o), group 7 and 8 received 3% Tween 20 and distilled water, while Misoprotol (100 mg/kg) was administered orally for the ninth group. After 1 h, all the animals were given 1 ml of absolute ethanol by oral gavage. One hour after the administration of ethanol, animals were sacrificed under ether anesthesia. The stomach of each rat was removed and opened along the greater curvature and washed in physiological saline solution. For the measurement of the gross gastric mucosal lesions, freshly excised stomach was laid flat and the mucosal lesions were then scored. The number and severity of erosions were scored using the scoring method as follows: 0 = No lesion, 0.5 = Hemorrhage, 1 = 1–3 small lesions < 10 mm length, 2 = 1–3 large lesions > 10 mm length, 3 = 1–3 thickened lesions, 4 = more than 3 small lesions, 5 = more than 3 large lesions, 6 = more than 3 thickened lesions [9]. The results were expressed as ulcer index (UI). The percentage protection was also calculated using the following formula:

$$\text{Pr}\text{otection}\left(\%\right)=\frac{\text{UI control}-\text{UI treated group}\;}{\text{UI control}}\;x\;100$$

### Indomethacin - induced model

The experiment was carried out according to the method of Ezike et al. [10] with a few modifications. Forty five fasted animals were used in nine groups of five animals each. Groups 1–6 (FPL and FPR treated group) received 100, 200 and 400 mg/kg of the extracts (p.o), group 7 and 8 received 3% Tween 20 and distilled water, while misoprotol (100 mg/kg) was administered orally for the ninth group. After one hour, all animals received indomethacin 40 mg/kg orally. The rats were sacrificed with ether anesthesia after six hours of drug treatment. The stomachs were isolated, washed gently with normal saline and cut open along the greater curvature. Mucosa lesions were then scored as previously mentioned.

### Pylorus ligation (PL)-induced ulcers

Drugs were administered for a period of 5 days by oral gavage and the rats were kept for 18 h fasting [11]. Animals were anaesthetized using urethane (5 ml/kg, i.p.), the abdomen was opened and pylorus ligation was done without causing any damage to its blood supply. The animals were deprived of water during the post-operative period [12]. After 6 h, stomachs were dissected out and cut open along the greater curvature and ulcers were scored in the glandular portion of the stomach. The volume of gastric juice (ml) and pH values were determined. The total acid secretion in the gastric juice supernatant was determined by titration to pH 7.0, using a 0.01 N NaOH solution, and phenolphthalein as indicator.

### Statistical analysis

The results were analyzed using GraphPad Prism version 5.0 software and Student’s *t*-test was employed for comparing the means between groups. The level of significance was set at p < 0.05.

## Results

### Phytochemical analysis

Preliminary analysis of the chemical composition of FPL and FPR extracts revealed the presence of terpenoids, tannins and saponins while alkaloid was not detected in both extracts. Flavonoid was detected only in the leaf, while anthraquinone was present in the root and not in the leaf.

### Acute toxicity test

At higher doses, most of the animals showed increased respiratory rate, hypoactivity, loss of appetite, and dyspnea before death. The oral acute toxicity study produced LD_50_ values of 4570 and 2754 mg/kg p.o. for FPL and FPR respectively at 24 h. In respect of the i.p. route, no death was recorded between 50 and 100 mg/kg while mortality was 100% at the highest dose of 2000 mg/kg for both FPL and FPR. The LD_50_ was estimated to be 1202.26 and 1380.38 mg/kg i.p. for FPL and FPR respectively.

### Effect on acute gastric mucosal lesions induced by ethanol

The effect of orally administered FPL and FPR on gastric damage induced by absolute ethanol is shown in Table 1. Oral administration of absolute ethanol to the control animals (3% Tween 20 for FPL and distilled water for FPR) produced multiple mucosal lesions in the rat stomach in the form of hemorrhagic streaks with ulcer index of 3.00 ± 0.81 and 4.8 ± 0.73 respectively.

**Table 1:** **Effect of *F. paniculata *leaf (FPL) and root (FPR) extracts on ethanol-induced gastric ulcers**

**Treatment**	**Dose (mg/kg)**	**Ulcer index**	**% Protection**
Control (3% Tween 20)	-	3.00 ± 0.81	-
FPL	100	0.53 ± 0.37^*^	82.22
	200	1.25 ± 0.63^*^	58.33
	400	1.73 ± 1.24	42.22
Control (water)	-	4.8 ± 0.73	-
FPR	100	3.67 ± 0.67	23.61
	200	3.8 ± 0.62	20.83
	400	3.6 ± 0.81	25.00
Misoprostol	100	0.3 ± 0.68 ^*^	93.75

The results are mean ± SEM for 5 animals/group. * Significantly different from the control at p < 0.05.

FPL (100, 200 and 400 mg/kg) inhibited ulcer formation by 82, 58 and 42% respectively, with the 100 mg/kg dose showing the highest inhibition which is statistically (p < 0.05) different from control (Table 1). FPR (100, 200 and 400 mg/kg) produced a weaker inhibition of gastric lesion with % inhibition of 23, 20 and 25 respectively. The inhibition among these dose groups was not statistically significant (p > 0.05). Misoprostol, the positive control, also offered significant protection (93% inhibition) against ethanol induced gastric lesions and the effect was higher than that of FPL and FPR.

### Effect on acute gastric mucosal lesions induced by indomethacin

The treatment with FPL (100, 200 and 400 mg/kg) and misoprostol (100 mg/kg) reduced the ulcer index compared with the control (Table 2). Similar to ethanol model, a significant protection (67%) (p < 0.05) was recorded for FPL at 100 mg/kg. FPR on the other hand demonstrated very weak activity with no inhibition recorded for 200 and 400 mg/kg doses.

**Table 2:** **Effect of *F. paniculata *leaf (FPL) and root (FPR) extracts on indomethacin induced gastric ulcer**

**Treatment**	**Dose (mg/kg)**	**Ulcer index**	**% Protection**
Control (3% Tween 20)	-	5.1 ± 0.56	-
FPL	100	1.67 ± 1.08^*^	67.32
	200	2.5 ± 1.14	50.98
	400	2.33 ± 0.49^*^	54.24
Control (distilled water)	-	2.92 ± 0.71	-
FPR	100	2.08 ± 0.96	28.57
	200	4.5 ± 0.82	0.00
	400	3.08 ± 0.95	0.00
Misoprostol	100	0.2 ± 0.48 ^*^	93.14

The results are mean ± SEM for 5 animals/group. * Significantly different from the control at p < 0.05.

### Effect of FPL extract on pylorus ligation induced gastric ulcers

The data on antiulcerogenic activity of both FPL and FPR in ethanol and indomethacin induced models suggest that FPR failed to reduce the gastric lesions induced by these models; therefore, FPR was discontinued with.

The effect of five day treatment with FPL and cimetidine on pylorus ligation induced gastric lesion in rats is shown in Table 3. Additional data concerning the volume, pH and acidity of the gastric juice is also presented. Pretreatment with FPL showed a dose-dependent antiulcer effect which was significant (p < 0.05) only at 400 mg/kg dose when compared with 3% tween 20 treated group. FPL-treated group (100 and 200 mg/kg) demonstrated significant reduction (p < 0.05) in the volume of gastric acid secretion. Total acidity of gastric juice was also reduced, though not statistically significant. There was no significant alteration (p > 0.05) in pH of both the extract and cimetidine.

**Table 3:** Effect of FPL extract on pylorus ligation induced gastric ulcers

**Treatment**	**Dose (mg/kg)**	**Ulcer index**	**% Inhibition**	**Gastric juice (ml)**	**Gastric pH**	**Gastric acidity (mEq/l)**
Control (3% Tween 20)	-	1.81 ± 0.5	-	0.95 ± 0.11	2.89 ± 0.28	11.04 ± 1.76
FPL	100	1.19 ± 0.26	34.7	0.72 ± 0.21	2.61 ± 0.41	7.17 ± 0.61
	200	0.69 ± 0.17	61.9	0.50 ± 0.13*	2.64 ± 0.19	7.30 ± 0.75
	400	0.37 ± 0.25*	79.6	0.22 ± 0.05*	3.10 ± 0.21	6.30 ± 1.41
Cimetidine	100	0.62 ± 0.42	42.9	0.86 ± 0.18	3.22 ± 0.33	9.6 ± 0.71

The results are mean ± SEM for 5 animals/group. * Significantly different from the control at p < 0.05.

## Discussion

Peptic ulcer is a very prevalent gastrointestinal disorder, characterized by disruption of the mucosal integrity attributed to various aggressive factors (acid, pepsin, stress, *Helicobacter pylori,* and NSAIDs) and defensive factors (mucus, bicarbonate, blood flow and prostaglandins) [13]. Different therapeutic agents including plant extracts are used to inhibit the gastric acid secretion or to boost the mucosal defense mechanism by increasing mucus production, stabilizing the surface epithelial cells or interfering with the PGs synthesis [14]. This study compares the antiulcer activity of ethanolic extracts of FPL and FPR in ethanol and indomethacin models. Ethanol induced model is used to screen drugs for cytoprotection while indomethacin-induced ulcer model shows both cytoprotection and gastric acid secretion [15]. Furthermore, the effect of FPL in pylorus ligation model was evaluated. This study is the first documentation on the antiulcer activity of this plant.

Ethanol is considered one of the agents that induce more intense gastric ulcers because it promotes serious disturbances in the gastric mucosa [16]. Ethanol induces ulcers by the reduction of gastric mucosal blood flow and mucus production in the gastric lumen, a decrease in endogenous glutathione and prostaglandin levels and an increase of ischemia, gastric vascular permeability, acid ‘back diffusion’, histamine release, efflux of sodium and potassium, influx of calcium, generation of free radicals and production of leukotrienes [17]. In this model, FPL reduced the development of ethanol induced gastric ulcers, with activity highest at a dose of 100 mg/kg. The inhibition was 82%, while misoprostol showed 93% ulcer inhibition when compared to control. This observation indicates gastric cytoprotective effect of FPL. FPR on the other hand failed to inhibit ethanol induced gastric ulcers in rats.

Non-steroidal anti-inflammatory drugs like indomethacin are commonly used pharmacological agents. They induce gastric lesions due to the reduction of endogenous prostaglandin synthesis which is known to be cytoprotective in the gastric mucosa [18]. Compared to the control, administration of FPL inhibited ulcerative lesion, with the highest inhibition recorded for 100 mg/kg dose while FPR extract did not show any significant activity in this model. This observation shows that FPL extract may have protected the gastric mucosa by enhancing prostaglandin synthesis and confirmed the cytoprotective effect of FPL extract.

Considering the data obtained from ethanol and indomethacin ulcer models, the observation that the response of the extracts decreased with increased dose is consistent with findings of studies on some other plants as reported by other authors [19-22]. Such a phenomenon of less response at higher dose is not uncommon with indigenous plants and this observation could be due to ‘therapeutic windows’ effect as suggested by Tripathi [23] and Zakaria et al. [24]. In other words, it could be that, the effect of the extract has reached its maximum level at a dose of 100 mg/kg, the lowest dose, used in this study.

Pylorus ligation is an important procedure that shows the possible changes of the parameters for gastric content e.g. volume of gastric juice, total acidity and pH [25]. Ulcers caused by pyloric ligation are due to increased accumulation of gastric acid and pepsin, leading to the autodigesion of gastric mucosa [26]. Inhibition of gastric acidity is one of the important protective factors, since overwhelming of the mucosal defense mechanisms by acid level leads to ulcer formation [27]. In this model, only 400 mg/kg of the extract exerted a significant reduction in ulcer index when compared with the control group. FPL exerted a significant preventive antiulcer effect in the pylorus-ligated model as indicated by reduction in gastric volume at 200 and 400 mg/kg doses. These results suggest that the extract interfered with digestive effect of accumulated gastric juice. Gastric acid secretion is stimulated by histamine release from enterochromaffin-like cells in the oxyntic glands; gastrin, released from G cells in the pyloric gastric glands and by acetylcholine, released from postganglionic enteric neurons [28]. The reduction of gastric acidity and gastric secretory volume could be attributed to antihistamine effect, since antihistamine drugs like cimetidine blocks H_2_ receptors in the stomach thereby reducing the acidity of the gastric juice [27].

The oral route LD_50_ of FPL was estimated to be 4570 mg/kg while that of FPR was 2754 mg/kg. The LD_50_ in intraperitoneal injection was estimated to be 1202.26 and 1380.38 mg/kg for FPL and FPR respectively. Based on the toxicity rating chart [29] and Hodge and Stemer scale [30]; the extracts could be classified as being slightly toxic by oral treatment and intraperitoneal injection. Although acute toxicity is a short term study and it does not document any gross organ toxicity, hence, there is the need to carry out a long term toxicity study of the extracts.

The presence of terpenoids, tannins and saponins was detected in FPL and FPR extracts while flavonoids were only present in FPL. Barros et al. [31] reported that phenolic compounds have an antiulcerogenic effect related to cytoprotective activity. Moreover, flavonoids such as quercetin have been reported to prevent gastric mucosal lesions in various experimental models by increasing the amount of neutral glycoproteins [32,33]. Flavonoids have been reported to protect the gastric mucosa from damage by increasing the mucosal prostaglandin content and by inhibiting histamine secretion from mast cells by inhibition of histidine decarboxylase [34]. Saponins and triterpenoids have been reported to have antiulcer activity in several experimental models by the formation of protective mucus on the gastric mucosa and also protect the mucosa from acid effects by selectively inhibiting prostaglandin [35,36]. Also, tannins have been shown to precipitate microproteins at the site of ulcers thereby forming an impervious protective pellicle over the lining to prevent adsorption of toxic substance thereby preventing the attack of proteolytic enzyme. These plant constituents present in FPL might be responsible for the observed activity.

## Conclusion

The present study indicated that FPL was more effective against experimentally induced gastric ulcer models. This may justify its use and validated the inclusion of the plant in folk preparations for gastrointestinal remedies in the South Western part of Nigeria. These results provide basis for further studies on FPL. Detailed histological assessment will be part of the follow-up study on FPL, including the bioactivity-guided fractionation to determine the bioactive principle(s) in the plant.

## Competing interests

The authors declare that they have no competing interests.

## Authors' contributions

All authors contributed equally in data acquisition. MOS drafted the manuscript. All authors read and approved the final manuscript.

## Pre-publication history

The pre-publication history for this paper can be accessed here:

http://www.biomedcentral.com/1472-6882/12/168/prepub
